# Does the MDS-UPDRS provide the precision to assess progression in early Parkinson’s disease? Learnings from the Parkinson’s progression marker initiative cohort

**DOI:** 10.1007/s00415-019-09348-3

**Published:** 2019-05-09

**Authors:** Antoine Regnault, Babak Boroojerdi, Juliette Meunier, Massimo Bani, Thomas Morel, Stefan Cano

**Affiliations:** 1 Modus Outcomes, 61 Cours de la Liberté, 69003 Lyon, France; 2 UCB Biosciences, Monheim, Germany; 3 UCB Biopharma Sprl, Braine-l’Alleud, Brussels, Belgium; 4 Modus Outcomes, Suite 210b, Spirella Building, Letchworth Garden City, SG6 4ET UK

**Keywords:** Early Parkinson’s disease, Movement Disorder Society Unified Parkinson’s Disease Rating Scale, Disease-modifying therapy, Rasch measurement theory

## Abstract

**Objectives:**

Developing disease modifying therapies for Parkinson’s disease (PD) calls for outcome measurement strategies focused on characterizing early stage disease progression. We explored the psychometric evidence for using the Movement Disorder Society Unified Parkinson’s Disease Rating Scale (MDS-UPDRS) part II (patient motor experience of daily living) and part III (clinician motor examination) in this context.

**Methods:**

MDS-UPDRS-II and -III data were collected at screening, month 12, and month 24 from 384 early stage PD patients (diagnosis ≤ 2 years; Hoehn and Yahr stage 1/2) in the Parkinson’s Progression Markers Initiative (PPMI) study. Psychometric analysis, based on Rasch measurement theory (RMT), was performed on both the original MDS UPDRS-II and -III scales and exploratory content-driven scale structures.

**Results:**

RMT analyses showed neither scale was well targeted to early PD. A marked floor effect appeared for most items and a clear item gap was consistently observed in very mild severity of motor signs and levels of motor impact. The original MDS-UPDRS-II and -III scales also displayed disordered thresholds (9/13 and 20/33 items, respectively), indicating response scales not functioning as expected, and misfit (5/13 and 12/33 items, respectively), flagging areas for potential improvement.

**Conclusions:**

The MDS-UPDRS-II and -III have psychometric limitations which limits the precision of measurement of motor symptoms and impact in early PD. This can lead to insensitivity in detecting differences and clinical change. Importantly, the diagnostic psychometric evidence provided by the RMT analysis provides a clear starting point for how to improve the quantification of clinically relevant concepts to characterize the course of early PD.

**Electronic supplementary material:**

The online version of this article (10.1007/s00415-019-09348-3) contains supplementary material, which is available to authorized users.

## Introduction

Several recent studies have grappled with the question of how to characterize the progression of Parkinson’s disease (PD). Studies have included estimations of the rate of progression [12] and explored the impact of laterality on the progression of disease [5]. Others have examined progression patterns in potentially different typologies of PD patients such as tremor dominant and postural instability/gait difficulty phenotypes of PD [17] or tried to determine whether baseline PD subtypes can serve as clinically useful predictors of disease progression rate [18].

However, any research on the progression of PD hinges on how we define PD severity and the methods we use to measure severity once it has been defined. For example, when we examine the rate of progression of PD, the key question is how we can best quantify the change (i.e., through imaging results, clinician rated severity rating, patient self-report). The underpinning question of the measure of the severity of PD is, therefore, fundamental in this context.

In order for research on the progression of PD to be truly useful, especially in the context of clinical trials, we need to assess PD progression using measurement techniques that allow accurate and meaningful assessment across the broadest range of severity and the broadest time frame possible on the same overall metric. In other words, we need to be able to examine disease progression from the early stages of very mild PD to the most advanced and severe disease on the same overall ruler. Measuring severity in the early, least severe stages of PD is particularly important when considering how to assess and demonstrate the benefits of new therapies for PD, especially disease-modifying therapies (DMT), where a key therapeutic objective is to postpone progression of PD from its early stages.

The continuing development and use of imaging biomarkers such as DaT-SPECT imaging with (123I) ioflupane single-photon emission computed tomography imaging offers a promising avenue for examining disease progression [4]. However, the meaningfulness of these measures is not necessarily straightforward. For example, Simuni and colleagues found the correlation of available imaging outcomes with clinician ratings of severity was weak [16], raising questions about their meaningful interpretation. It is also unclear how these imaging outcomes translate in terms of direct patient experience.

An alternative approach to measuring PD severity is to rely on rating scales, based on clinician or patient reports. The Movement Disorder Society-sponsored revision of the Unified Parkinson's Disease Rating Scale (MDS-UPDRS) is undoubtedly the most widely used rating scale in PD [7, 8] and is endorsed by the Movement Disorder Society (MDS) as the recommended rating scale to measure PD disability.

However, though MDS-UPDRS is widely used in PD, there is limited published evidence regarding its measurement performance in early stages of the disease. Therefore, it is unclear to what extent the MDS-UPDRS is appropriate for studying progression in PD, especially when examining progression from the early stages of the disease. This lack of evidence around MDS-UPDRS performance in early disease also calls into question whether it is an appropriate outcome measure to use for clinical development of DMTs.

Our objective was to explore the psychometric evidence for using two components of the MDS-UPDRS that relate to motor signs in the context of early PD: MDS-UPDRS-II (13 items) and MDS-UPDRS-III (33 items), which assess patient-reported impact of motor signs of PD and clinician-reported severity of motor signs of PD, respectively.

## Methods

### Study sample: the Parkinson Progression Markers Initiative (PPMI)

The Parkinson Progression Markers Initiative (PPMI) Study 001 is an ongoing longitudinal, international, multicentre study designed to assess progression of clinical features, imaging and biologic markers in Parkinson disease patients (de novo PD, prodromal, and patients with specific genetic mutations) and healthy controls. It is conducted by PPMI, a collaboration of researchers, funders and study participants working toward the goal of identifying progression biomarkers to improve PD therapeutics and is sponsored by The Michael J. Fox Foundation for Parkinson’s Research (https://www.ppmi-info.org/about-ppmi/).

The study plans to include approximately 2000 patients in 6 cohorts: PD (400 individuals), patients who have scans without evidence of dopaminergic degeneration (i.e., SWEDD, 70 individuals), prodromal (100 individuals), a genetic cohort of PD patients and unaffected individuals (600 individuals), a genetic registry (600 individuals), and healthy controls (200 individuals). The expected follow-up will range between three and eight years. The study includes series of clinical assessments (including the MDS-UPDRS), safety assessments, imaging, and biologic sampling.

Our analyses were performed on data from a data cut on January 11, 2016, using MDS-UPDRS-II and -III data collected at three timepoints (screening, month 12, month 24) from 384 early PD patients (diagnosis 2 years or less; Hoehn and Yahr stage I/II [11]). All analyses were performed using the MDS-UPDRS assessment off medication, more than six hours after the last dose of dopaminergic therapy as defined in the PPMI study protocol.

### Rasch measurement theory analysis

Rasch measurement theory (RMT) analyses use a mathematical model (the Rasch model) to evaluate the legitimacy of summing items to generate measurements [2, 10, 15]. An RMT analysis examines the extent to which the observed data (i.e. subjects’ actual responses to scale items) ‘fit’ predictions of those responses from the Rasch model, which in essence defines how a set of items should perform to generate reliable and valid measurements. The difference between expected and observed scores indicates the degree to which rigorous measurement is achieved. RMT analysis explores the following:

- Reliability: Reliability is assessed using the Person Separation Index (PSI) [1], a reliability coefficient estimate. Reliability coefficients are interpreted as follows: < 0.70: unsatisfactory; 0.70–0.79: modest; 0.80–0.89: adequate; 0.90–1.00: good (recommended in a high-stake decision context, such as the demonstration of new treatment efficacy) [14].
- Targeting: Scale-to-sample targeting concerns the match between the range of the target concept measured by the item set and the range of target concept in the sample of patients. This is assessed by examining the spread of person and item locations in these two relative distributions. This analysis informs us how suitable the sample is for evaluating the item set and how suitable the item set is for measuring the sample.
- Fit: Items must work together (fit) to define a clinically and statistically meaningful score. Otherwise, it is inappropriate to sum item responses to reach a total score and consider the total score an accurate measure of each target concept. When items do not work together in this way (i.e., there is item misfit), the validity of an item set is questionable. Evidence for item fit is based on natural ordering of item response options (ordering of item thresholds) [3], statistical indicators (standardized fit residual, chi-square), and graphical indicators (item characteristic curve; ICC) [19]. As a rule of thumb, standardized fit residual values are recommended to lie in the range − 2.50 to + 2.50 [10].
- Dependency: The response to any item in the item set should not directly influence the response to any another in the item set [13]. If this happens, measurement estimates may be biased, and reliability may be artificially elevated. RMT determines this effect by examining residual correlations.

RMT analyses were carried out using RUMM 2030 software (RUMM Laboratory, Perth, Australia) on the original MDS-UPDRS-II and -III scales as well as alternate exploratory content-driven scale structures.

For the MDS-UPDRS-III, items that are rated separately for the right and left side were specifically coded to reflect the asymmetrical course of PD in the RMT analysis. In other words, instead of having ‘left’ and ‘right’ ratings, our analysis specified ‘ipsilateral’ and ‘contralateral’ items, defined related to the predominant side of PD.

## Results

### Parkinson Progression Markers Initiative (PPMI) study sample

The majority of the 384 patients in this sample were male (*n* = 251, 65.4%). The mean age was 62 years, with a broad distribution across the four age groups identified. Most patients had been diagnosed for less than six months. Patients were divided relatively evenly between Hoehn and Yahr stages 1 and 2 (49% and 51%, respectively). These early stage patients reported little motor symptom interference with activities of daily living, with mean modified Schwab and England ADL [6] scores at 93.85 on a scale ranging from 0 to 100, where scores above 90 correspond to complete independence (Table 1).

**Table 1:** Baseline demographics and PD characteristics

Variable	PD cohort (*N* = 384)
Age, years	
Mean (SD)	62.02 (9.83)
Median	62.79
Age groups, *N* (%)	
≤ 55 years	94 (24.5%)
> 55 to 65 years	131 (34.1%)
> 65 to 70 years	71 (18.5%)
> 70 years	88 (22.9%)
Gender, *N* (%)	
Male	251 (65.4%)
Disease duration, years	
Mean (SD)	0.56 (0.57)
Median	0.34
Dominant side, *N* (%)	
Left	159 (41.4%)
Right	216 (56.3%)
Symmetric	9 (2.3%)
Hoehn and Yahr stage, *N* (%)	
Stage 1	188 (49.0%)
Stage 2	196 (51.0%)
Modified Schwab and England ADL, score	
Mean (SD)	93.85 (5.91)
Median	95.00

## MDS-UPDRS-III analyses

### Floor and ceiling effects, missing data

**Fig. 1:**
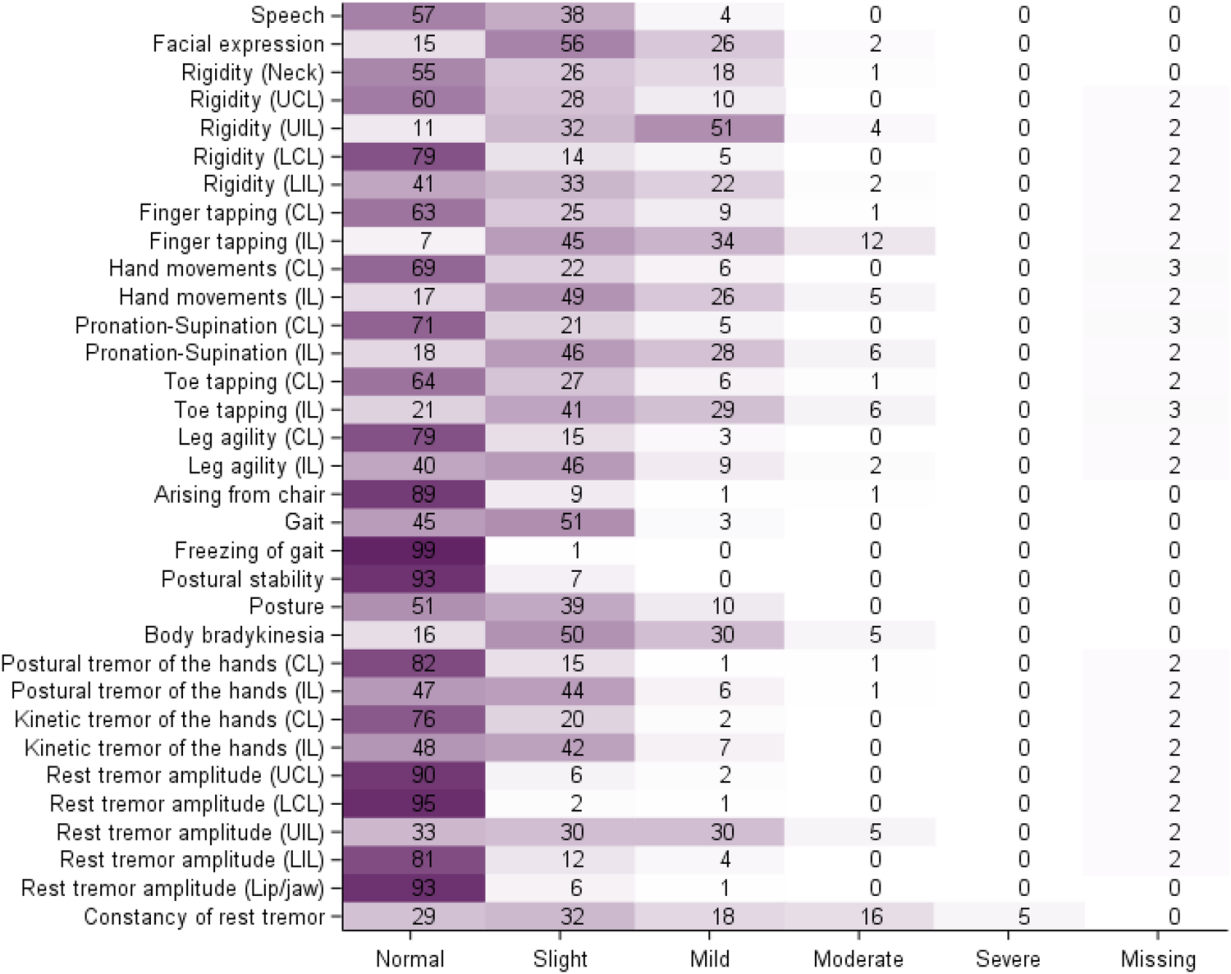
Heatmap of responses to MDS-UPDRS-III items (baseline PPMI PD cohort; *N* = 384). Each cell of the map shows the percentage of patients rated at the given level (column) for the given item (raw). Darker fill colours indicate higher percentages

The quality of data completion was good, with very little missing data. In this early PD sample, a floor effect was observed for responses to most UPDRS-III items (i.e., a substantial percentage of patients of the sample were rated as “Normal” for these signs), suggesting the items mostly reflect levels of severity of motor signs that are not experienced by these patients with early PD (Fig. 1).

### RMT analysis

We ran the Rasch model on responses from the 15 raw MDS-UPDRS-III items that do not assess right and left sides of the body separately and the 18 ‘lateralized’ items (transformed to reflect the predominant side of PD) using 1053 assessments (all non-extreme) of the UPDRS-III at screening, month 12 and month 24.

**Fig. 2:**
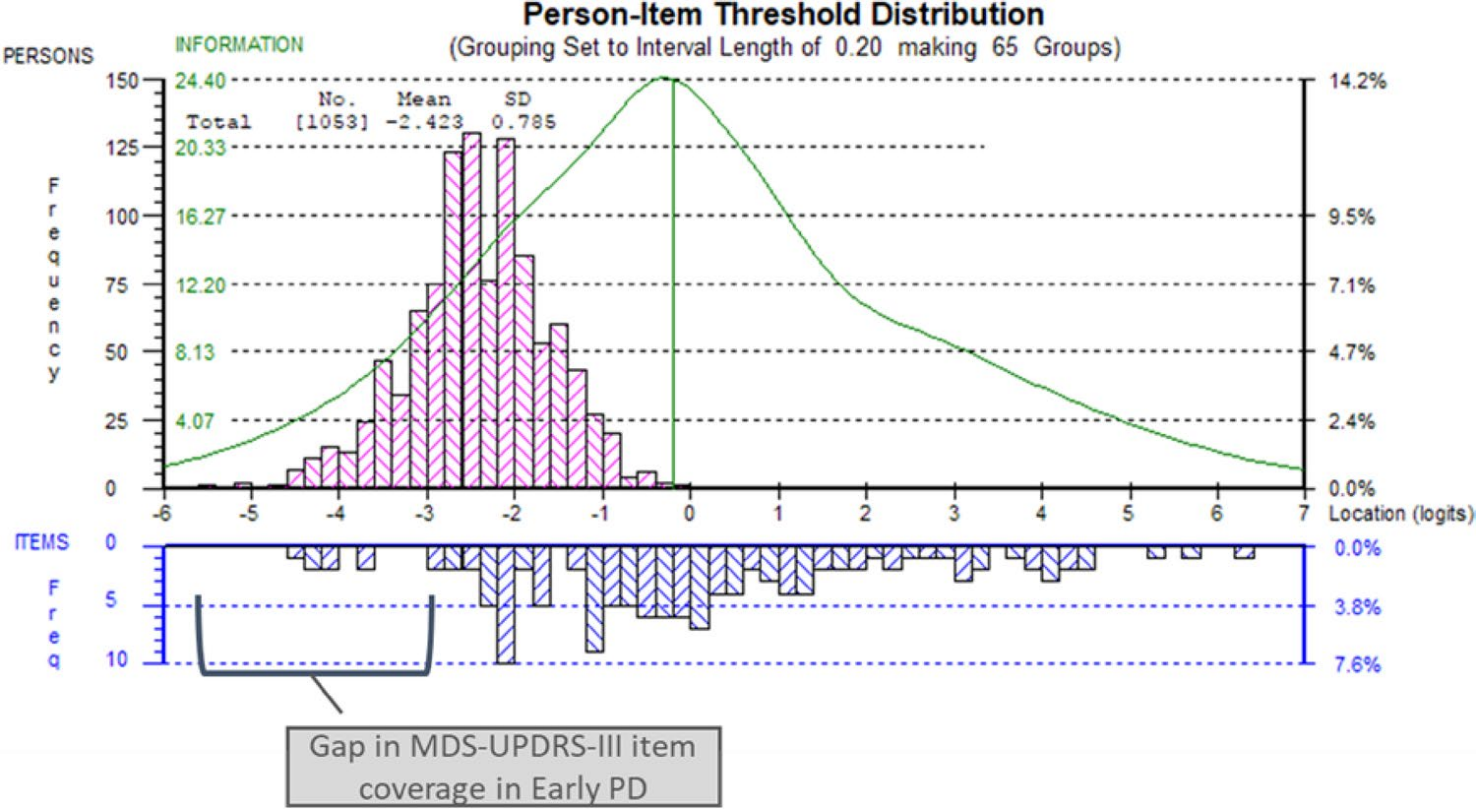
Scale to sample targeting of MDS-UPRS-III in the PPMI Parkinson’s disease cohort (screening, month 12, and month 24 pooled, *N* = 1053). The upper panel (pink boxes) shows the distribution of the individuals of the PPMI PD cohort over the continuum of motor sign severity; the lower panel (blue boxes) shows the distribution of the MDS-UPDRS-III items on the continuum of motor sign severity; the green line shows the information function of the MDS-UPDRS-III items, reflecting the accuracy of measurement over the continuum of severity of motor signs

These analyses indicated that the UPDRS-III items had adequate reliability (PSI: 0.86). However, analysis indicated that these items were mistargeted to the PPMI early disease sample (Fig. 2); in other words, our findings suggest that the UPDRS-III items largely reflect motor sign severity levels not experienced by patients of the PPMI early PD cohort during the two first years of follow-up.

In terms of fit, RMT analysis indicated that the items of the UPDRS-III item set broadly work together, with a clear and interpretable hierarchy of motor signs (Table 2). There is an apparent progression of item severity from unilateral bradykinesia and rigidity (upper extremity, then lower extremity), to midline functions (facial expression, posture and gait), bilateral bradykinesia and rigidity (as contralateral signs appear) and finally general movement issues (postural stability, rising from chair, freezing of gait). However, twelve misfitting items were detected (Table 2, in bold). The ‘constancy of rest tremor’ item showed clear misfit to the Rasch model (standard fit residual, 11.87). Evidence of misfit (i.e., standardized residuals out of the − 2.5/2.5 range) was found for several other tremor items, particularly ipsilateral tremor items. RMT analysis detected some issues with the response scales of the UPDRS-III items, as 21 items presented disordered thresholds. For most of these items, this result was not problematic as the thresholds not showing natural ordering were between greater severity categories and were not reliably estimated given the very few numbers of patients in the sample at this high severity level. The only items that showed disordered thresholds for milder motor signs were the items about rest tremor amplitude in upper and lower limbs (both ipsilateral and contralateral) and the item on consistency of rest tremors: for the former, the categories ‘Slight: ≤ 1 cm in maximal amplitude’ and ‘Mild: > 1 cm but < 3 cm in maximal amplitude’ (for the lower limb only) did not function as intended; for the later, it was the category ‘Mild: Tremor at rest is present 26–50% of the entire examination period’. These findings indicate possible issues experienced by clinicians with these ratings and these response categories may require further thoughtful examination.

**Table 2:** RMT analysis of MDS-UPDRS-III items with ipsilateral and contralateral categorization: presence of reversed thresholds, location estimates and fit statistics (PPMI Parkinson’s disease cohort screening, month 12, and month 24 pooled, *N* = 1053)

Item	Reversed thresholds	Location estimate (SE)	Standardized fit residual	*χ* ^2^	*p* value
18. Constancy of rest tremor	X	− 1.43 (0.03)	***11.87***	340.92	< 0.001
4. Ipsilateral Finger Tapping		− 1.24 (0.04)	− 1.61	14.81	0.0963
6. Ipsilateral Pronation-Supination		− 0.82 (0.04)	− 0.03	7.65	0.5700
5. Ipsilateral Hand movements		− 0.74 (0.04)	− ***2.99***	35.04	< 0.001
7. Ipsilateral Toe tapping		− 0.72 (0.04)	− 1.36	9.27	0.4126
14. Global spontaneity of movement		− 0.56 (0.04)	− ***3.09***	59.32	< 0.001
3c. Ipsilateral UE Rigidity		− 0.52 (0.05)	− 0.29	11.64	0.2342
2. Facial expression		− 0.49 (0.05)	− 1.80	27.08	0.0014
17. Ipsilateral UE rest tremor amplitude		− 0.41 (0.04)	***8.33***	192.83	< 0.001
3e. Ipsilateral LE rigidity		− 0.24 (0.04)	1.52	14.17	0.1163
8. Ipsilateral leg agility	X	− 0.21 (0.04)	− 2.43	15.98	0.0674
5. Ipsilateral Postural hand tremor	X	− 0.13 (0.05)	***3.21***	73.01	< 0.001
13. Posture	X	− 0.12 (0.05)	− 0.89	11.12	0.2679
3a. Neck rigidity		− 0.04 (0.04)	− 2.07	22.53	0.0073
10. Gait	X	− 0.01 (0.05)	− 0.12	2.33	0.9850
4. Contralateral finger tapping		0.00 (0.04)	− ***4.72***	60.41	< 0.001
16. Ipsilateral kinetic hand tremor	X	0.02 (0.05)	0.81	12.71	0.1763
17 Ipsilateral LE rest tremor amplitude	X	0.10 (0.05)	***3.75***	82.92	< 0.001
7. Contralateral toe tapping		0.13 (0.05)	− 1.88	28.43	< 0.001
1. Speech	X	0.22 (0.05)	− 0.31	6.46	0.6934
5. Contralateral hand movements	X	0.23 (0.05)	− ***5.48***	79.85	< 0.001
3c. Contralateral UE Rigidity	X	0.26 (0.05)	− ***3.45***	37.34	< 0.001
6. Contralateral Pronation-Supination	X	0.30 (0.05)	− ***4.91***	55.81	< 0.001
17. Contralateral LE rest tremor amplitude	X	0.30 (0.06)	2.25	14.05	0.1205
3e. Contralateral LE rigidity	X	0.34 (0.05)	− ***3.68***	39.55	< 0.001
12. Postural stability	X	0.39 (0.06)	1.28	13.43	0.1443
15. Contralateral postural hand tremor	X	0.45 (0.07)	0.35	9.17	0.4222
8. Contralateral leg agility	X	0.51 (0.06)	− ***4.31***	55.44	< 0.001
9. Arising from chair	X	0.52 (0.06)	− 1.30	4.83	0.8488
17. Contralateral LE rest tremor amplitude	X	0.67 (0.08)	1.26	20.77	0.0137
17e. Lip/jaw rest tremor amplitude	X	0.78 (0.09)	0.41	14.01	0.1221
16. Contralateral kinetic hand tremor	X	0.96 (0.07)	− 0.29	5.67	0.7723
11. Freezing of gait	X	1.50 (0.20)	− 1.94	11.72	0.2294

Items are ordered according to item location estimates to show the hierarchy revealed by the RMT analysis. Standardized fit residuals outside the recommended range − 2.5/ + 2.5 indicated in bold italics

Finally, potential local dependency between some items was indicated by high residual correlations; this typically occurred for items referring to similar sign clusters in the same side of the body [additional material].

## MDS-UPDRS-II analyses

### Floor and ceiling effects, missing data

**Fig. 3:**
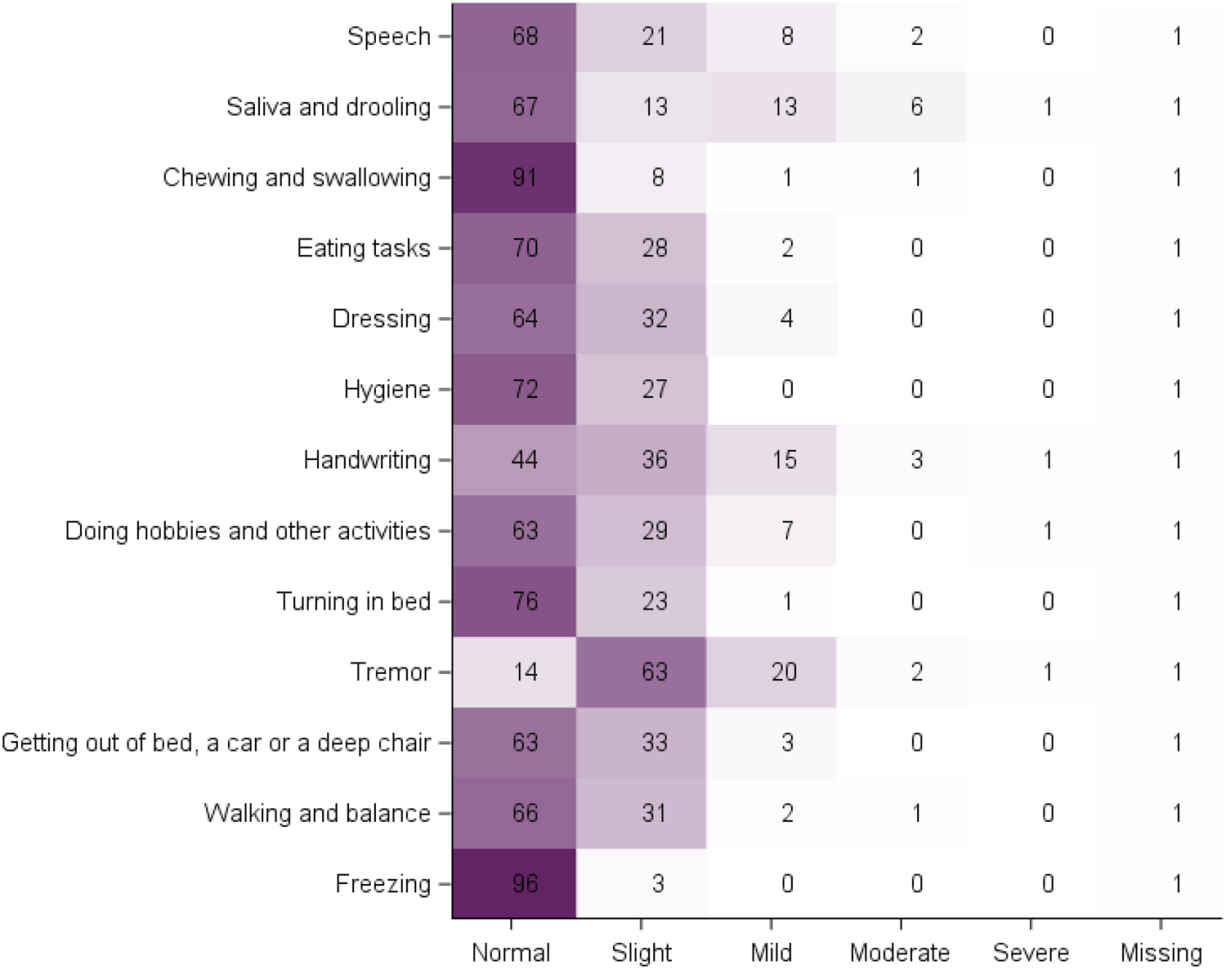
Heatmap of responses to the MDS-UPDRS-II items (baseline PPMI PD cohort; *N* = 384). Each cell of the map shows the percentage of patients rated at the given level (column) for the given item (raw). Darker fill colours indicate higher percentages

The MDS-UPDRS-II items were well completed with few missing data at each visit. A high floor effect was observed for all the items at baseline, month 12 and 24 with one exception: the “Tremor” item, for which < 15% of the PD patients answered “Normal” at each visit. The “Freezing” item had a particularly strong floor effect, with > 80% of patients answering “Normal” at each visit (Fig. 3).

Responses at screening, month 12, and month 24 for the original MDS-UPDRS-II items were subjected to RMT analysis. Reliability in this patient sample was modest (PSI 0.72).

**Fig. 4:**
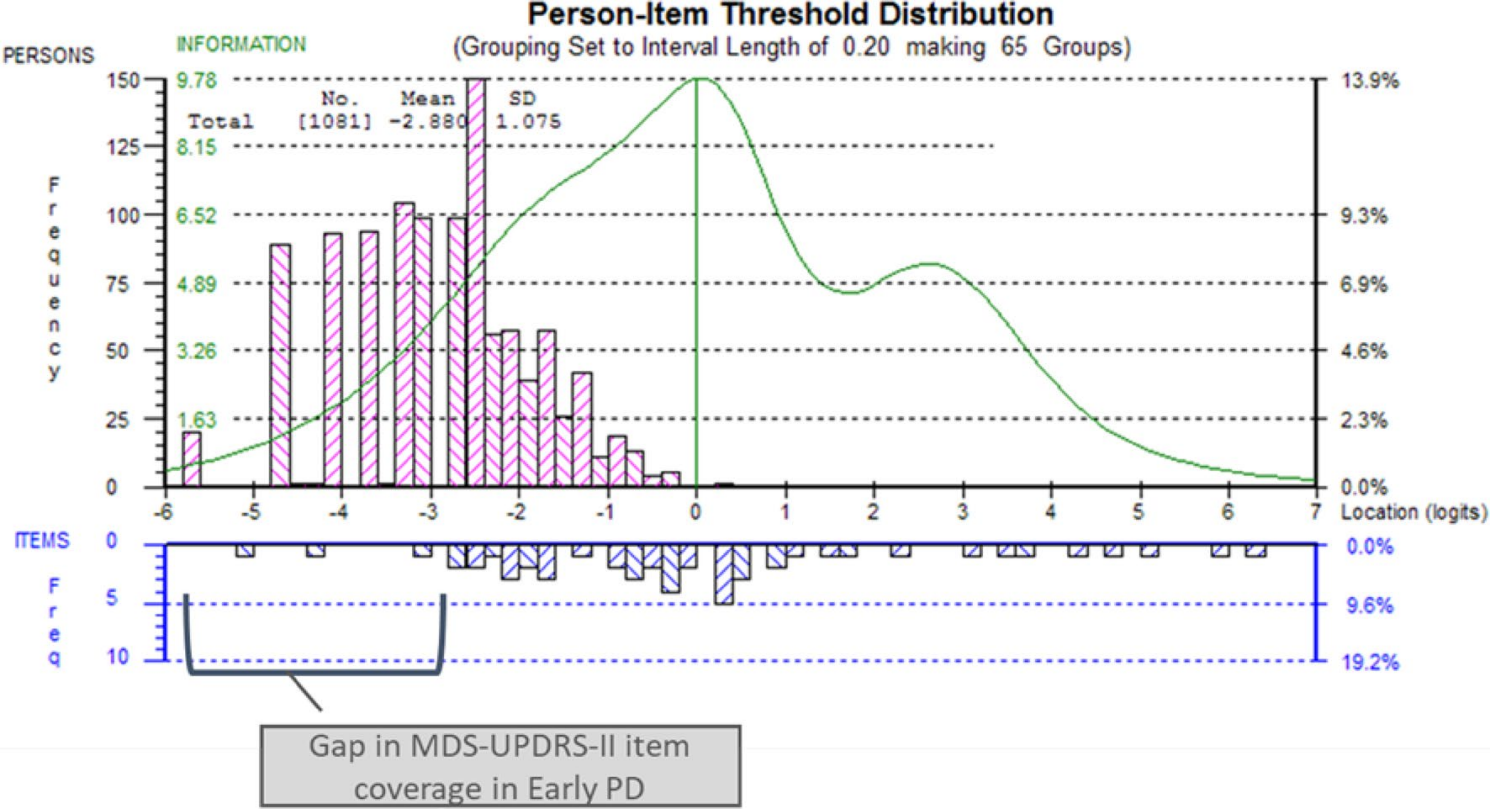
Scale to sample targeting of MDS-UPRS-II in the PPMI Parkinson’s disease cohort (screening, month 12, and month 24 pooled, *N* = 1081). The upper panel (pink boxes) shows the distribution of the individuals of the PPMI PD cohort over the continuum of impact of motor signs; the lower panel (blue boxes) shows the distribution of the MDS-UPDRS-II items on the continuum of impact of motor signs; the green line shows the information function of the MDS-UPDRS-II items, reflecting the accuracy of measurement over the continuum of impact of motor signs

Results indicated that MDS-UPDRS-II items were mistargeted to the PPMI patient sample. A large proportion of this early PD sample was not adequately covered by the MDS-UPDRS-II items; most item thresholds were characteristic of higher levels of the latent construct (i.e., items corresponded to high impact on ‘motor experience of daily living’) that are not relevant to the early PD patients of the PPMI sample (Fig. 4).

In terms of item fit, no clearly interpretable hierarchy of MDS-UPDRS-II items emerged from the RMT analysis. Seven of the 13 items demonstrated item misfit (Table 3). The ‘Tremor’ (under-discriminant; fit residual = 5.42), ‘Hygiene’ (over-discriminant, fit residual = − 4.38), ‘Dressing’ (over-discriminant; fit residual = − 4.83) and ‘Doing hobbies and other activities’ (over-discriminant; fit residuals = − 4.27) were the most problematic of these items in terms of fit. Further, nine of the 13 MDS-UPDRS-II items had disordered thresholds, indicating response options that patients could not distinguish appropriately. The main issues were for the items ‘Saliva and drooling’, ‘Chewing and swallowing’ and ‘Walking and balance’ for which response options reflecting fairly mild impact of motor symptoms (namely’ Slight: I have too much saliva, but do not drool’ ‘Mild: I need to have my pills cut or my food specially prepared because of chewing or swallowing problems, but I have not choked over the past week’ and ‘Mild: I occasionally use a walking aid, but I do not need any help from another person’ respectively) did not function as intended. Finally, no local dependency between items was observed, as no residual correlation was greater than 0.30 [Additional material].

**Table 3:** RMT analysis of the MDS-UPDRS-II items: presence of reversed thresholds, location estimates and fit statistics (PPMI Parkinson’s disease cohort screening, month 12, and month 24 pooled, *N* = 1053)

Item	Reversed thresholds	Location parameter estimate (SE)	Standardized fit residual	*χ* ^2^	*p* value
10. Tremor		− 1.50 (0.05)	***5.42***	214.71	< 0.0001
7. Handwriting		− 1.48 (0.04)	1.03	31.67	0.0002
2. Saliva and drooling	X	− 1.18 (0.04)	1.07	32.67	0.0002
8. Doing hobbies and other activities	X	− 0.82 (0.05)	− ***4.27***	50.85	< 0.0001
1. Speech		− 0.62 (0.04)	− 0.57	12.68	0.1775
13. Freezing	X	0.15 (0.10)	− ***2.77***	15.46	0.0791
12. Walking and balance	X	0.35 (0.06)	− 1.50	21.69	0.0099
11. Getting out of bed, a car or a deep chair		0.44 (0.06)	− ***3.26***	31.95	0.0002
5. Dressing	X	0.44 (0.06)	− ***4.83***	76.36	< 0.0001
3. Chewing and swallowing	X	0.69 (0.07)	− 0.82	5.69	0.7707
4. Eating tasks	X	1.06 (0.06)	− ***2.86***	25.40	0.0026
6. Hygiene	X	1.21 (0.07)	− ***4.38***	49.54	< 0.0001
9. Turning in bed	X	1.25 (0.07)	− 2.45	24.17	0.0040

Items are ordered according to item location estimates to show the hierarchy revealed by the RMT analysis Standardized fit residuals outside the recommended range − 2.5/ + 2.5 indicated in bold italics

We examined possible enhancement of the results by testing alternative coding for the response categories of items showing disordered thresholds and exclusion of the ‘Tremor’ item, but the alternate coding did not result in significantly better targeting or item fit in either case.

Because the results of these models were not satisfactory, and no clear hierarchy could be found in the MDS-UPDRS-II items, we explored a reconceptualization of the MDS-UPDRS-II items. We distinguished two different domains: “Function” (Speech, Saliva and drooling, Chewing and swallowing, Turning in bed, Tremor, Getting out of bed, a car or a deep chair, Walking and balance, Freezing), and “Daily Life” (Eating tasks, Dressing, Hygiene, Handwriting, Doing hobbies and other activities). However, the RMT analyses conducted on these two set of items still found mistargeting, misfitting items, unsatisfactory reliability and no obvious item hierarchy.

## Discussion

The MDS-UPDRS is an obvious candidate rating scale to be used for the characterization of PD progression; it is the most widely used in PD and is already used for this purpose. Two recent papers reported longitudinal analyses from the PPMI study [12, 16]. However, while these analyses use the MDS-UPDRS data, they do not address the question of whether measuring PD severity with this rating scale is appropriate in this specific context of use (i.e. early PD). To shed light on this key concern, we conducted a series of psychometric analyses of the two components of the MDS-UPDRS relating to motor signs (Part II and III) to better understand the scale’s performance in this context.

The PPMI data were particularly relevant for our objectives since it included MDS-UPDRS data collected in a cohort of de novo PD patients. To be included in this PD cohort, patients had to have been diagnosed with PD for within the last two years. We performed analyses on pooled data using baseline, year 1 and year 2 data; thus, this sample included patients within four years of diagnosis, a good representation of the breadth of early disease experience.

### MDS-UPDRS-III

Our analyses indicated that measuring motor signs of PD through the clinician-reported MDS-UPDRS-III in the early stages of PD may not be optimal, since most of the items correspond to more advanced features of the disease. Only a few items assess signs that are relevant to an early PD population; these include items regarding upper and lower extremity bradykinesia and rigidity and to a lesser extent, some midline functions (facial expression, speech, gait, posture). This lack of items in the lower spectrum of disease severity jeopardizes the scale’s ability to accurately discriminate between patients depending on the severity of their motor signs in early stages of PD.

However, the RMT analysis also demonstrated empirically that the MDS-UPDRS-III items reflect a meaningful clinical hierarchy. Location estimates place items assessing signs characteristic at PD diagnosis—slight unilateral bradykinesia first in upper extremities, followed quickly by lower extremity bradykinesia and slight rigidity of upper extremities—at the lower end of severity. Of note, slight impairment of facial expression is also among the first manifestations of the disease, as is reflected in our analysis. The hierarchy of items continues through slight impairment of speech, gait and posture; worsening of ipsilateral signs (bradykinesia, rigidity), with the addition of slight kinetic and postural tremors; and bilaterality of signs (slight contralateral bradykinesia and rigidity); concluding in items reflecting worsening of bilateral signs and balance impairment (postural stability, arising from chair). Thus, in this sense, the MDS-UPDRS-III is a promising basis for measuring the progression of PD, as it can be considered as a single metric across the severity continuum. However, to detect change and potential treatment benefit in early PD and DMT trials, more information on motor signs specific to early stages of PD (slight and mild bradykinesia, rigidity and tremor) will be needed to bridge the measurement gap at the milder severity end of the continuum. This information could come from revision of the current items (e.g., improving the rest tremor items that demonstrated problems in our analyses) or by obtaining a greater granularity of information for these items by adding new questions or modifying their response scales.

### MDS-UPDRS-II

The findings of the analysis of the MDS-UPDRS-II were more problematic and highlighted the limitations of this scale in measuring patient-reported motor signs and PD impact on patient daily life. Many psychometric issues arose during analysis, including mistargeting, misfit, inappropriateness of the response scales, and modest reliability. Importantly, these analyses confirmed that the scale lacks conceptual clarity; with items appearing to assess very different concepts, including symptoms (e.g., tremor), functions (chewing, swallowing), and daily activities (e.g., hobbies). We tested several alternative approaches to the MDS-UPDRS-II items, including scale reconceptualization, but none led to significant improvement in measurement performance. A previous RMT analysis of the part II of the original version of the UPDRS, in a more heterogenous sample of patients with PD (i.e., not including early PD only), uncovered very similar measurement limitations and attributed them to issues with the conceptual underpinning of the UPDRS-II [9]. Thus, we must conclude that different rating scales may be more appropriate for assessing patient-reported motor symptoms and impact of PD than MDS-UPDRS-II in early PD, but maybe also in the range of disease severity.

### Limitations

Our analysis focused only on the assessment motor signs of PD through MDS-UPDRS-II and III. It is possible that characterizing PD progression requires the assessment of non-motor aspects as well; this may be particularly the case for the early stages of disease where motor signs are much less apparent.

### Future research

Our findings suggest that additional research is needed to address measurement gaps present in MDS-UPDRS-II and III. While the clinician-reported MDS-UPDRS-III offers a sound basis for examining disease progression in PD patients, determining how to address the conceptual gaps in the measurement of motor signs is important to ensure that the rating scale can detect progression and change in the less severe early PD population. Further research could help determine if there are better ways to capture early motor signs of PD, if there are motor signs that are not included in the UPDRS-III that are relevant to the early PD population, or if there are ways to provide for greater granularity in how the signs currently included are assessed. Given the measurement limitations of the MDS-UPDRS-II, we advocate for exploring alternative methods to measure the patient perception of motor signs and their impact in early PD, grounded in a thorough, evidence-based understanding of the key aspects of motor sign symptom and impact for patients in the early stages of the disease. Research in partnership with early PD patients is best suited to achieve that goal.

## Conclusion

The MDS-UPDRS-II and -III have psychometric limitations which limits the precision of measurement of motor symptoms and impact in early PD. In particular, mistargeting of many items suggests the scales may not be sensitive in terms of detecting differences and clinical change. This constitutes important limitations for the use of the MDS-UPDRS-II and -III in the context of clinical trials of DMT. However, the item hierarchy of the clinician-reported MDS-UPDRS-III items revealed by the RMT analysis offers a clear and clinically interpretable frame of reference that highlights the area of possible improvement towards a better measure of progression of early PD.

## Electronic supplementary material

Below is the link to the electronic supplementary material.

Supplementary file1 (XLSX 26 kb)
